# Synergy or Independence? Deciphering the Interaction of HLA Class I and NK Cell KIR Alleles in Early HIV-1 Disease Progression

**DOI:** 10.1371/journal.ppat.0030043

**Published:** 2007-04-20

**Authors:** Jason D Barbour, Uma Sriram, Stacy J Caillier, Jay A Levy, Frederick M Hecht, Jorge R Oksenberg

**Affiliations:** The Scripps Research Institute, United States of America

## Introduction

Individual susceptibility to infectious disease, such as HIV-1, is strongly influenced by the genetic profile of the host. Allelic variants of the human major histocompatibility complex (human leukocyte antigen [HLA]) genes have been implicated repeatedly with susceptibility, course, and outcome of HIV-1 infection [1]. The HLA Class I loci is genetically diverse, and in addition to its functions in presenting peptide fragments of the invading pathogen to CD8+ T cells, may assume other functions, such as direct interaction with natural killer (NK) cells. Alleles of the Class I A and B loci form the *Bw4/Bw6* cluster, a grouping based on an exposed epitope on the α1 helix, on the lip of the peptide binding cleft. This region of the HLA molecule is known to interact with NK cells via the killer Ig-like receptor (KIR). Expressed on the NK cell surface, KIR is allelic, with forms that, upon engagement with an HLA Class I ligand, may either inhibit or activate an NK cell. The KIR receptor alleles fall into two broad groups: forms with a long cytoplasmic tail (such as *KIR2DL1*) bear an immune tyrosine inhibitory motif and inhibit, while the short-tail forms (such as *KIR2DS2*) activate NK cells [2]. Upon engagement with an HLA Class I ligand, short-tail forms of KIR may increase NK cell effector activity, inducing the release of perforin and granzyme, and secretion of IFN-γ [2] .

## Summary of Observational Studies on HLA and KIR on HIV-1 Disease Progression

Flores-Villanueva and colleagues noted that HIV-1-infected persons with superior control of viral replication in the absence of anti-retroviral treatment were more likely to be homozygous for a *Bw4* allele than those who could not control infection [3]. The *Bw4* cluster, in addition to bearing an epitope that interacts with certain KIRs, contains a number of HLA types known to confer protection against HIV-1 disease progression, such as B*57 and B*27. The *Bw4* cluster does not contain the B*35 allele, of which the B*35Px variant [4] has been linked to accelerated disease progression. Hence, the association of *Bw4* with improved outcomes may be linked to the inclusion of strongly protective alleles such as B*57, the exclusion of others such as B*35, and or interaction with a form of *KIR*.

The *Bw4* epitope contains a subset of alleles known as *Bw4Ile80,* which bear an isoleucine in position 80 of the α1 helix, the lip of the HLA binding cleft. The *Bw4Ile80* epitope may be important in T cell modulation [5] and immunomodulation of NK cell function [6]. The Bw4Ile80 epitope interacts with the KIR3DL1 inhibitory allele. KIR3DS1 is a short-tailed form of KIR, and has very high sequence homology to KIR3DL1 in its extracellular domains. KIR3DS1 was predicted to likewise interact with Bw4Ile80 molecules, although this interaction had not been directly observed. Martin et al. examined the epistatic interaction of *KIR3DS1* and *Bw4Ile80* for the effect on time to the onset of AIDS [7] in a cohort of HIV-1-infected adults in North America. They observed that *Bw4Ile80/KIR3DS1* carriers experienced a significant delay in time to disease. The authors controlled their analysis for the known effects of the protective B*27, B*57, and the deleterious B*35 alleles. Hence, the *Bw4Ile80/KIR3DS1* effect on disease progression may be due to a synergistic benefit of carriage of both the *Bw4Ile80* and *KIR3DS1* alleles, an effect which may be attributable to enhanced NK cell activity via *KIR* activation [2].

In a subsequent study by Qi et al. [8] based on same cohorts of HIV-1-infected persons studied by Martin et al., *Bw4Ile80/KIR3DS1* was associated with a delayed risk of development of certain opportunistic infections. Qi et al. observed that *Bw4Ile80/KIR3DS1* carriers had an average HIV-1 RNA level of 4.75 log_10_ copies/mL versus a viral load of 4.89 log_10_ copies/mL for those who did not carry both genes, and suggested this difference might at least partially explain the observed clinical benefits. This difference in viral replication is modest, and may not be sufficient to explain the benefit in long-term clinical outcome in HIV-1 disease that was observed among persons with *Bw4Ile80/KIR3DS1*.

Gaudieri et al. examined HLA and KIR effects in a cohort of HIV-1-infected persons in Australia [9]. The authors observed an increased relative rate of CD4+ T cell percent loss for those carrying *KIR3DS1*. In contrast to results by Martin et al., Gaudieri et al. did not find a significant protective effect against CD4+ T cell loss among *Bw4Ile80/KIR3DS1* carriers, and in fact found these persons were at increased hazard (relative hazard of 14) for reaching AIDS (under the 1987 US Centers for Disease Control and Prevention definition).

In our contemporary cohort of recently HIV-1-infected persons, we had the opportunity to observe the association of *Bw4Ile80* and *KIR3DS1* alleles on HIV-1 RNA and CD4+ T cell levels during very early infection (see Protocol S1). These individuals were treatment-naïve adults with precisely known dates of seroconversion (within six months of seroconversion for 90% of cases). Of the 255 persons in this analysis, the median HIV-1 RNA level at study entry was 4.66 (interquartile range [IQR] 3.77, 5.29) log_10_ HIV-1 RNA copies/mL, and the median CD4+ T cell count was 534 (IQR 410, 674) cells/uL. These persons were observed for a total of 263.7 person-years prior to initiating potent anti-retroviral therapy. The study population was 88% Caucasian and 94% were male, reflecting the HIV-1 epidemic in San Francisco, where we are based. Of these 255 individuals, 94 (37%) carried at least one *KIR3DS1* allele, 239 (94%) carried at least one *KIR3DL1* allele, 114 (45%) carried at least one HLA Class I B *Bw4Ile80* allele, and 43 (17%) carried both a *KIR3DS1* and an HLA-B *Bw4Ile80* allele. We considered only HLA Class I B alleles as part of the *Bw4Ile80* cluster for this analysis.

**Figure 1:**
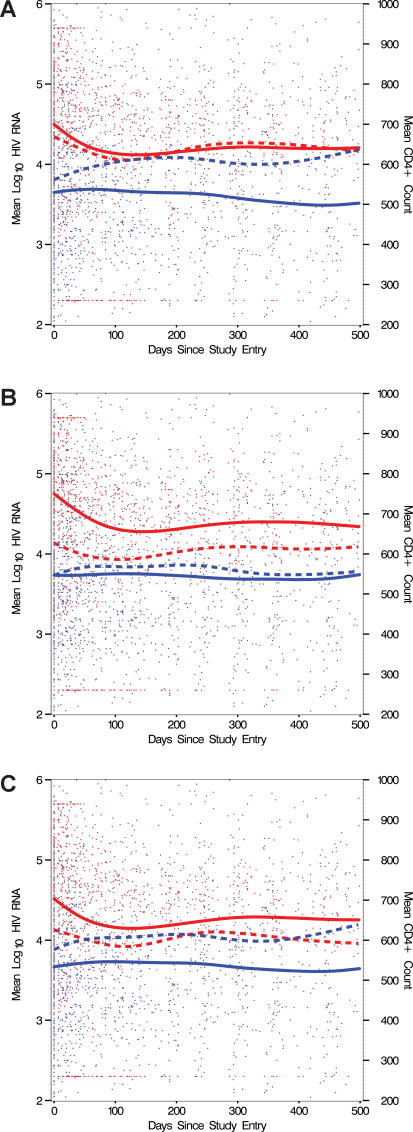
Effects of *Bw4Ile80* and *KIR3DS1* Gene Carriage on HIV Disease Outcome (A) Those with *KIR3DS1* (dashed line) versus those without *KIR3DS1*. The dashed red line represents a moving average of HIV-1 RNA levels over time among those carrying at least one *KIR3DS1* allele. The solid red line represents those that do not carry the *KIR3DS1* allele. The dashed blue line represents a moving average of CD4+ T cell counts over time among those carrying at least one *KIR3DS1* allele. The solid blue line represents those that do not carry the *KIR3DS1* allele. (B) Those with *Bw4Ile80* (dashed line) versus those without *Bw4Ile80*. The dashed red line represents a moving average of HIV-1 RNA levels over time among those carrying at least one *Bw4Ile80* allele. The solid red line represents those that do not carry the *Bw4Ile80* allele. The dashed blue line represents a moving average of CD4+ T cell counts over time among those carrying at least one *Bw4Ile80* allele. The solid blue line represents those that do not carry the *Bw4Ile80* allele. (C) Those with both the *Bw4Ile80* and *KIR3DS1* alleles (dashed line) versus those without both alleles. The dashed red line represents a moving average of HIV-1 RNA levels over time among those carrying both the *Bw4Ile80* and *KIR3DS1* alleles. The solid red line represents those that do not carry both the *Bw4Ile80* and *KIR3DS1* alleles. The dashed blue line represents a moving average of CD4+ T cell counts over time among those carrying at both the *Bw4Ile80* and *KIR3DS1* alleles. The solid blue line represents those that do not carry both the *Bw4Ile80* and *KIR3DS1* alleles.

With the benefit of multiple HIV-1 RNA and CD4+ T cell measures from very early in infection, we observed that the *KIR3DS1*-bearing individuals had higher CD4+ T cell counts during early infection (average + 58 cells/uL [standard error (SE) = 28.5] higher over time, *p* = 0.04, Figure 1A), but did not have lower HIV-1 RNA levels (−0.11 [SE = 0.12] log_10_ copies/mL, *p* = 0.34, Figure 1A). In contrast, those carrying the *Bw4Ile80* allele had a significant reduction in viral load at study entry (−0.37 log_10_ [SE = 0.10] copies/mL, *p* = 0.0003, Figure 1B), but did not display a significant difference in CD4+ T cell counts (−1.5 [SE = 25.5] cells/uL, *p* = 0.95, Figure 1B). Those carrying both *Bw4Ile80* and *KIR3DS1* did not display a significant trend towards higher CD4+ T cell counts over the period of observation (+ 40 cells/uL [SE = 36.5], *p* = 0.27, Figure 1C), and displayed a trend towards a reduction in viral loads (−0.22 log_10_ [SE = 0.15] lower HIV-1 RNA copies/mL, *p* = 0.14, Figure 1C) relative to those who did not carry both genes.

The reduction in viral load among *Bw4Ile80/KIR3DS1* carriers was smaller (−0.22 log_10_ [SE = 0.15] copies/ml, *p* = 0.14; Figure 1C) than that reduction seen for all *Bw4Ile80* (−0.37 log_10_ copies/mL, *p* = 0.0003, Figure 1B). And, the effect on CD4+ T cell count was smaller and not statistically significant for *Bw4Ile80/KIR3DS1* compared to the effect for *KIR3DS1* alone (+ 40 cells/uL versus + 58 cells/uL, Figure 1C versus 1A). That neither the viral load nor CD4+ T cell effects were augmented among carriers of *Bw4Ile80/KIR3DS1* suggests that *Bw4Ile80* and *KIR3DS1* do not synergize to confer greater protective effects on HIV-1 disease markers in early infection, a period in which innate immunity ought to be active.

To further explore the possibility of synergy between *Bw4Ile80* and *KIR3DS1,* we compared those with *KIR3DS1* and lacking *Bw4Ile80* to those who carried both genes. If *Bw4Ile80* and *KIR3DS1* were operating synergistically, we would expect to see a greater benefit on HIV-1 RNA levels, or CD4+ T cell counts, among *Bw4Ile80/KIR3DS1* versus those carrying *KIR3DS1* alone. The *Bw4Ile80/KIR3DS1* carriers did not display a trend towards higher CD4+ T cell counts (−0.10 [SE = 48.3] cells/uL over time, *p* = 0.9) versus those who carried *KIR3DS1* alone. Those with both *Bw4Ile80* and *KIR3DS1* demonstrated a marginally significant trend towards viral load reductions (−0.29 [SE = 0.19] log_10_ copies/mL, *p* = 0.12) versus those who carried *KIR3DS1* alone. This magnitude of difference in viral load was similar to the deficit observed between those who did and did not carry *Bw4Ile80* (Figure 1B), regardless of *KIR3DS1* carriage (which was −0.37 log_10_ copies/mL, Figure 1B). This suggests that the effect on viral load reduction was due to the *Bw4Ile80* gene itself, and not due to an interaction with *KIR3DS1*. Indeed, when persons bearing B*57 (a *Bw4Ile80* allele known to confer lower viral loads) were removed from the analysis, the magnitude of the viral load reduction was reduced from −0.37 log_10_ copies/mL to −0.25 (SE = 0.10) log_10_ copies/mL, *p* = 0.02). The HLA B*57 allele, which is known to associate with very low viral loads during early infection, may confer a large share of the viral load reduction among *Bw4Ile80* carriers, regardless of *KIR3DS1* carriage. Taken together, these data suggest that *KIR3DS1* may associate with elevated CD4+ T cell counts during early infection in a manner independent of viral load, with *Bw4Ile80* having a modest additive benefit via a small reduction in HIV-1 RNA levels.

The reduction in viral load that we observed for the *Bw4Ile80/KIR3DS1* group was slightly larger than that observed by Qi et al. (−0.14 log_10_ c/ml for Qi et al. versus our observation of −0.22 log_10_ c/mL). This magnitude of difference in HIV-1 RNA levels observed by *Bw4Ile80/KIR3DS1* is small and may not be sufficient to explain differences in CD4+ T cell levels. Our results indicate that there is an effect of *KIR3DS1* carriage on CD4+ T cell counts independent of *Bw4Ile80* carriage in early HIV-1 infection. *Bw4Ile80/KIR3DS1* joint carriage was associated with a modest reduction in HIV-1 RNA levels, which is likely due to independent activity of *Bw4Ile80* member alleles, such as B*57. The effect of *KIR3DS1* on CD4+ T cell counts during early infection may reflect higher CD4+ T cell counts prior to acquisition of infection, a sparing of CD4+ T cells during early infection, or both.

We performed our analyses considering only HLA Class I B loci alleles as part of the *Bw4Ile80* cluster. This is consistent with the approach by Martin et al. [7] and facilitates comparison of results. When we included HLA Class I A loci alleles as part of the *Bw4Ile80* cluster, our main inference—the absence of synergy between *Bw4Ile80* and *KIR3DS1* on either viral load or CD4+ T cell count—was unchanged.

Until recently, it has not been clear whether HLA Class I A loci alleles, which bear a *Bw4Ile80* motif, interact with KIR alleles, such as *KIR3DL1*. Recent work has suggested that certain HLA Class I A *Bw4Ile80* alleles, such as A*24, interact with differing *KIR3DL1* allotypes [10]. Binding of A*2402 to certain allotypes was influenced by the nature of the peptide bound. HIV-1 Nef and p17 peptides were observed to enhance A*2402 binding to certain *KIR3DL1* allotypes. Hence, sequence of the acquired viral strain, and peptide presentation patterns within the infected person, may influence the interaction of HLA and KIR molecules.

Recent experimental results may shed light on the question of Bw4 and KIR3DS1 interaction. O'Connor et al. recently verified that KIR3DS1 is in fact expressed on the NK cell surface, and was higher among homozgyotes than heterozygotes. However, Carr et al. [11] and O'Connor et al. [12] each separately demonstrated that neither *Bw4* nor *Bw6* ligands recognize KIR3DS1 extracellular domains. KIR3DS1 extracellular domains bear high sequence homology to *KIR3DL1* and were predicted to be recognized by Bw4 and Bw4Ile80 molecules. That it was not recognized may indicate that Bw4Ile80 molecules do not, as a group, recognize KIR3DS1. Alternatively, and as the authors suggest, it is possible that KIR allotype recognition by a Class I A or B Bw4Ile80 molecule is dependent on the peptide bound in the HLA cleft [10]. But, that has yet to be shown for KIR3DS1.

Our study focused on correlates of HIV-1 RNA and CD4+ T cell levels in the earliest stages of HIV-1 infection. Disease progression rates are established in early infection [13,14], and innate immune activity such as NK cell activity ought to be present in the earliest stages of infection. An NK cell–mediated effect on disease parameters such as CD4+ T cell counts or viral load is likely to be observed at that time. The study by Martin et al. noted that the effect of *Bw4Ile80/KIR3DS1* carriage on disease outcome (the fraction remaining AIDS-free) was not present until year three of infection. A definition of clinical AIDS is reached at a time of severe CD4+ T cell loss late in infection. Hence, there may have been an observable effect on CD4+ T cell count and rate of loss or on elevated and increasing HIV-1 RNA levels during earlier time periods in the Martin et al. cohort.

The studies by Martin et al. and Qi et al. take a survival analysis or time-to-event approach to an historical cohort with extended pre-treatment follow-up. This approach to observational data does not make use of all available clinical data. In our cohort, we used longitudinal mixed effects modeling statistics (as did Gaudieri et al.) that allowed us to make use of all of our data over time. Our cohort has the advantage of being contemporary (enrollment 1996 to present) and having tightly spaced HIV-1 RNA and CD4+ T cell measurements during early infection. Our study is larger in enrollment than that by Gaudieri et al., and has more precisely known dates for seroconversion. However, our cohort, as it has been under observation since the advent of effective anti-retroviral therapy, has less untreated follow-up than either of these prior studies. Therefore, our data do not extend to the effect of *Bw4Ile80* and *KIR3DS1* carriage beyond the first two years of HIV-1 infection.

Neither our study nor others described here have addressed the issue of *KIR* “dose” or expression patterns beyond whether an individual is an homozygotic or heterozygotic carrier of an allele. That is, individuals carry variable numbers of *KIR* genes (six to 19 genes) [2], which can lead to complex expression patterns. The number and pattern of *KIR3DS1* or other activating alleles carried by an individual may have an impact on the clinical phenotypes described here, and may help explain the discrepancy in outcomes across studies.

Alternative explanations for a role of *KIR3DS1* in delayed disease progression may exist. KIR molecules may be found on cell types other than NK cells. For example, KIR2DS2 molecules are expressed on T cells [15] among those suffering from chronic inflammatory conditions such as rheumatoid arthritis [16], and may influence the activation state and cytokine expression of these T cell populations. Snyder et al. observed that, upon antibody stimulation of surface KIR2DS2 on CD4+ CD28^null^ DAP12–expressing T cells, the signaling protein Erk was phosphorylated and IFN-γ production increased [17]. In this way, *KIR*-activating alleles, of which KIR3DS1 is a member, may influence CD4+ and/or CD8+ T cell activation states in a manner independent of T cell receptor stimulation. Hence, it is possible the KIR3DS1 expression on lymphocytes influences the activity and activation state of memory CD4+ T cells in HIV disease and, in turn, influences bystander cell death or dysfunction of uninfected CD4+ T cells [18]. Again, the ligand for such an effect is not known.

While our data do not support an interaction for Bw4Ile80 and KIR3DS1, it remains possible that there is an interaction which manifests later in disease, dependent on an unknown ligand or on presentation of specific HIV-1 peptide fragments that are not processed until later in infection. Alternatively, *KIR3DS1* expression alone may confer a protective effect on CD4+ T cell counts in HIV-1 disease absent interaction with a Bw4 ligand via direct modulation of CD4+ or CD8+ T cells that express KIR [17]. Further experimental work is needed to verify the regulation, expression, and localization of *KIR3DS1,* its interaction with Class I ligands, and dependence on the peptide fragment bound by a Class I molecule. 

## Supporting Information

Protocol S1 Materials and Methods (38 KB DOC)

## Abbreviations

HLA: human leukocyte antigen
KIR: killer Ig-like receptor
NK: natural killer
SE: standard error
