# Correction: Synergy or Independence? Deciphering the Interaction of HLA Class I and NK Cell KIR Alleles in Early HIV-1 Disease Progression

**DOI:** 10.1371/journal.ppat.0030154

**Published:** 2007-10-26

**Authors:** Jason D Barbour, Uma Sriram, Stacy J Caillier, Jay A Levy, Frederick M Hecht, Jorge R Oksenberg

Correction for: Barbour JD, Sriram U, Caillier SJ, Levy JA, Hecht FM, et al. (2007) Synergy or independence? Deciphering the putative interaction of HLA Class I and NK cell KIR receptor alleles on early HIV-1 disease progression. PLoS Pathog 3(4): e43. doi: 10.1371/journal.ppat.0030043

In *PLoS Pathogens,* volume 3, issue 4:

The funding statement for the above paper was incomplete. The full funding statement follows.

**Funding.** This work was supported by grants from the National Institutes of Health (NIH 1UO1 AI41531 to JAL and FMH, and NIH, Brodsky P01-AI064520). JDB is supported by K01 AI 066917 (National Institute of Allergy and Infectious Diseases/NIH).

