# Supplementary material for: Synergy or Independence? Deciphering the Interaction of HLA Class I and NK Cell KIR Alleles in Early HIV-1 Disease Progression
Source: PLoS Pathog. 2007 Apr 20;3(4):e43. doi: 10.1371/journal.ppat.0030043 (PMC1853116; doi:10.1371/journal.ppat.0030043)
Supplement: Supplementary file 1 [file ppat.0030043.sd001.doc]

**Materials and Methods:**

**Study Subjects:** Subjects with possible recent HIV-1 infection were recruited through a broad community network that is maintained through regular outreach and identified as being in early HIV-1 infection through HIV-1 antibody and HIV-1 RNA testing as previously described [1]. Among those enrolled, approximately 90% were within 6 months of acquiring HIV-1 infection. Patients were followed regardless whether they elected early antiretroviral treatment or remained off treatment. The majority of patients (60%) elected to receive antiretroviral therapy within 1 year of acquiring HIV-1 infection. Subjects were included in this analysis if HLA and KIR genetic testing were available. All subjects provided informed consent and this study was approved by the Committee on Human Research at the University of California, San Francisco.

**Laboratory Methods:** Molecular typing of *HLA*-*A, -B* loci was performed using a non-radioactive PCR based sequence specific oligonucleotide probe reverse line-blot assay (PCR-SSOP) (Dynal, Norway). Genotyping for killer inhibitory receptor *KIR3DS1* and *KIR3DL1* was performed as described by Uhrberg *et al* [2]. Genomic DNA from cell line CF996 was used as a positive control in each PCR for *KIR3DS1*[3].

**Statistical Methods:** We right censored persons from the analysis at the time they initiated an anti-retroviral regimen or were lost from follow-up. We employed a mixed effects model [4], with random effects for time and intercept, to assess differences in log_10_ HIV-1 RNA and absolute CD4+ T cell count over time. We constructed models to test for differences in rate of change, as well as difference in average level, for both outcome measures by membership in the *BwIle80/KIR3DS1* allele categories. Average levels are reported in all cases here. All statistical analyses were performed in SAS Version 9.2 (SAS Institute, Cary, NC, USA) for Windows XP.

References

1. Hecht FM, Busch MP, Rawal B, Webb M, Rosenberg E, et al. (2002) Use of laboratory tests and clinical symptoms for identification of primary HIV infection. Aids 16: 1119-1129.

2. Uhrberg M, Parham P, Wernet P (2002) Definition of gene content for nine common group B haplotypes of the Caucasoid population: KIR haplotypes contain between seven and eleven KIR genes. Immunogenetics 54: 221-229.

3. Cook MA, Norman PJ, Curran MD, Maxwell LD, Briggs DC, et al. (2003) A multi-laboratory characterization of the KIR genotypes of 10th International Histocompatibility Workshop cell lines. Hum Immunol 64: 567-571.

4. Diggle P, Liang, K., Zeger, S. (1994) Analysis of Longitudinal Data. Oxford: Oxford University Press.
